# Classification of Interstitial Lung Abnormality Patterns with an Ensemble of Deep Convolutional Neural Networks

**DOI:** 10.1038/s41598-019-56989-5

**Published:** 2020-01-15

**Authors:** David Bermejo-Peláez, Samuel Y. Ash, George R. Washko, Raúl San José Estépar, María J. Ledesma-Carbayo

**Affiliations:** 1 Biomedical Image Technologies, ETSI Telecomunicación, Universidad Politécnica de Madrid & CIBER-BBN, Madrid, Spain; 2 Applied Chest Imaging Laboratory, Department of Radiology, Brigham and Women’s Hospital, Boston, Massachusetts, United States of America; 3 Division of Pulmonary and Critical Care Medicine, Department of Medicine, Brigham and Women’s Hospital, Boston, MA USA

**Keywords:** Biomarkers, Translational research, Computer science

## Abstract

Subtle interstitial changes in the lung parenchyma of smokers, known as Interstitial Lung Abnormalities (ILA), have been associated with clinical outcomes, including mortality, even in the absence of Interstitial Lung Disease (ILD). Although several methods have been proposed for the automatic identification of more advanced Interstitial Lung Disease (ILD) patterns, few have tackled ILA, which likely precedes the development ILD in some cases. In this context, we propose a novel methodology for automated identification and classification of ILA patterns in computed tomography (CT) images. The proposed method is an ensemble of deep convolutional neural networks (CNNs) that detect more discriminative features by incorporating two, two-and-a-half and three- dimensional architectures, thereby enabling more accurate classification. This technique is implemented by first training each individual CNN, and then combining its output responses to form the overall ensemble output. To train and test the system we used 37424 radiographic tissue samples corresponding to eight different parenchymal feature classes from 208 CT scans. The resulting ensemble performance including an average sensitivity of 91,41% and average specificity of 98,18% suggests it is potentially a viable method to identify radiographic patterns that precede the development of ILD.

## Introduction

Chronic cigarette smoke exposure is associated with a variety of effects on the lung parenchyma. While there are other, more uncommon manifestations, generally, these effects can be characterized as those associated with destruction of the parenchyma leading to a local decreases in lung density typically called emphysema, and those associated with inflammation and scar formation leading to local increases in lung density, typically called as interstitial changes. In fact, these manifestations can co-exist even within the same individual.

Interstitial Lung Diseases (ILD) are a heterogeneous group of more than 200 lung disorders that largely affect the lung parenchyma but which may present airway or vascular manifestations as well. There is a growing acceptance that some forms of ILD, especially idiopathic pulmonary fibrosis (IPF) may be preceded by early or subtle radiographic findings seen on computed tomography (CT) scans of the chest^1,2^. The visual presence of these findings, often termed interstitial lung abnormalities (ILA) has been shown to be associated with reduced lung volume, increased mortality and a genetic polymorphism that is also associated with IPF^3–7^. The rapid growth in use of CT scanning, combined with the high mortality associated with IPF and the fact that its available treatments slow but do not reverse the disease process, has led to a growing need for methods to accurately detect early interstitial changes before they progress to end stage fibrosis^8,9^. While visual CT analysis remains the mainstay of clinical imaging interpretation, there has been an increasing recognition of the potential role of objective analysis techniques to quantify abnormalities on CT and characterize disease subtypes^10^.

**Figure 1:**
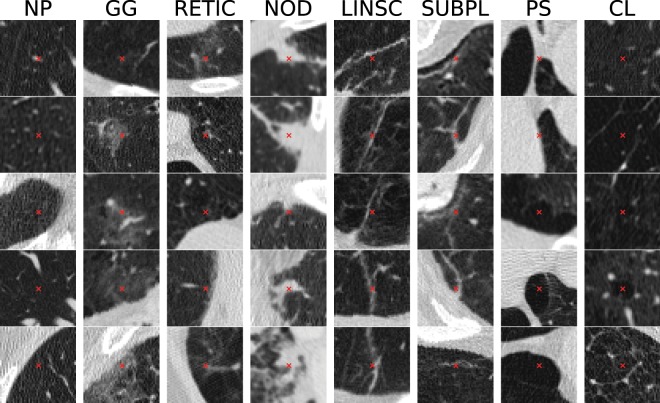
Examples of patches centered on selected points along different ILA patterns, including normal parenchyma (NP), ground-glass (GG), reticular (RETIC), nodular (NOD), linear scar (LINSC), subpleural line (SUBPL), paraseptal emphysema (PS) and centrilobular emphysema (CL).

In this paper we propose a method for objective and automated identification and classification of ILA and emphysematous patterns on chest CT scans, based on Convolutional Neural Networks (CNNs), considering a total of eight different radiographic patterns: normal parenchyma, five interstitial patterns (ground-glass, reticular, nodular, linear scar, subpleural line) and two emphysematous patterns (centrilobular and paraseptal) as depicted in Fig. 1.

### Related work

There have been several approaches to computer-aided analysis and automated identification and classification of ILD using CT images. For example, in patients with IPF, densitometric measures, such as the skewness, kurtosis and the mean of the histogram of distribution of lung density, have been found to be associated with outcomes such as pulmonary function and transplant free survival^11,12^. Hand-crafted texture features such as gray level co-occurrence matrices (GLCM), run length matrices (RLM), and fractal analysis, have been also widely used^13–15^ for classification of patterns of interstitial change as have local binary patterns (LBP)^16^ and local histogram-based measures^10,17^. For example, Park *et al*. proposed a CAD scheme to detect, but not classify, early ILD using low-dose CT images based on hand-crafted features such as RLM and histogram features on 30 patients^18^.

More recent approaches have proposed the use of features learned directly from the data, instead of hand-crafted ones. Some of these methods are based on unsupervised learning algorithms such as restricted Boltzmann machine (RBM)^19^ or k-means and k-SVD to construct a set of learned features^20,21^.

Multiple deep learning approaches have been used to prognosticate and characterize disease. Convolutional Neural Networks (CNN) have been trained for a number of purposes, including characterization of disease severity and prediction of clinical outcomes^22^, pulmonary artery-vein separation^23^, biomarker regression^24^, pulmonary fissure detection^25^ or emphysema classification^26,27^ using CT data from the COPDGene study, a large multi-center study with over 10,000 subjects.

For ILD characterization, there has been proposed a CNN-based method for automatic classification of patients with fibrotic lung disease^28^, as well as a method for classifying the image-level label of ILD types^29^. There has also been an attempt to classify in an holistic manner whole lung slices by using a pre-trained and fine-tuned AlexNet architecture^30^. However, a holistic classification of a whole slice as a unique label does not consider multiple tissue subtypes in the same slice thus it only provides a rough quantification method of the disease. Regarding ILD radiographic subtype classification, other recent notable approaches include the use of a variety of CNNs. These include CNNs to classify 2D patches as interstitial patterns with or without pre-training using a variety of texture datasets^31–33^ as well as the use of the well-known architectures such as AlexNet and GoogleNet pre-trained on ImageNet^34^.

One potential approach for improving the performance of all of these techniques is to combine multiple classifiers into an ensemble. While this has not been attempted for ILD classification, others have used this approach for the identification of other lung structures such as nodules. These efforts have included both the combination of multiple CNN with the same configuration, each of them having as input a different nodule view^35^ and the combination of MLP, KNN and SVM to classify hand-crafted features for nodule diagnosis^36^.

In this work we propose the first deep learning-based method to identify and classify radiographic patterns of ILA, which likely represents early or subtle ILD in some cases, that implies the characterization of 8 different parenchymal features types. We propose a methodology based on an ensemble of various 2D, 2,5D and 3D CNN specific architectures to tackle subtle parenchymal patterns, including multi-context and multi-stage architectures, proving the superiority with respect to previous methods specifically designed to address late-stage interstitial diseases revealing the need of specific designs and research to tackle ILA properly. Additionally, this work precisely defines how to perform and optimize the ensemble of different CNN architectures which has rarely been addressed.

## Methods

### Database

The CT scans used in this study for both training and evaluating the proposed method were acquired as part of the COPDGene study, a previously described^37^ multi center study designed to identify genetic and epidemiologic factors associated with COPD. The COPDGene study was approved by Partners Human Research Committee (Protocol Number 2007-P-000554/2) and institutional review boards at all study sites (see Supplementary Table S1), and written informed consent was obtained from all subjects. All research in this study was performed in accordance with relevant guidelines and regulations.

The scans utilized for this study were acquired at full inspiration and were obtained with a total of 9 CT scanner models from 3 manufactures. From each CT scan, images were acquired using two different reconstruction kernels, namely a soft filter (B35, Smooth Recon) and a shaper one (B50, Sharp Recon). Two pulmonologists manually placed a total of 37427 training points in the scans of 208 randomly selected individuals. These points were placed throughout the lungs of the participants and included the following parenchymal features types: normal parenchyma, interstitial features including ground glass, reticular, nodular, linear scar and sub pleural line, and emphysematous features including centrilobular and paraseptal emphysema (Table 1). This list of features was felt to represent the majority of parenchymal tissue types in the cohort and all of the feature types were included, so that, during the classification process, the entire lung volume could be classified as a particular subtype. That is, no volume was classified as other or unclassifiable. Of note, normal parenchyma was labeled both distant from and adjacent to the other tissue subtypes, but only in areas that were clearly visually normal. Additionally, no pan-lobular emphysema was identified in the training cohort, likely due to the lack of alpha-1-antitrypsin disease, and thus this feature was excluded.

**Table 1:** Description of the database.

ILA radiographic pattern	Num. Points	Num. Scans
Normal parenchyma	23696	161
Ground-glass	137	9
Reticular	5409	58
Nodular	116	7
Linear scar	195	8
Paraseptal emphysema	3845	43
Centrilobular emphysema	3613	51
Subpleural line	413	19

### Multi-model ensemble

In order to address the problem of radiographic ILA pattern classification from CT images, we designed a multi-model ensemble of deep convolutional neural networks. Ensemble strategies allow combining the individual predictions of a set of classifiers following some defined criterion such as weighted averaging or majority voting rule. The combination of multiple independent models usually performs better than the independent models separately^38^. Individual networks work well on finding locally optimal solutions of the true function that maps the input image space to the output label space. Ensemble methods show their advantage by starting the optimal solution search of the true function from different locally optimal points. The ensemble algorithm can then combine different information from each individual model to construct a better representation of the input therefore enabling a better mapping function.

In the proposed ensemble method, each individual network is trained from scratch using the same training samples. Then, the output probability distribution responses of the networks are combined using a weighted averaging to form the overall output of the ensemble:

1 $${\rm{Y}}({\rm{x}})=\mathop{\sum }\limits_{{\rm{i}}=1}^{{\rm{n}}}{{\rm{w}}}_{{\rm{i}}}{{\rm{P}}}_{{\rm{i}}}({\rm{x}})$$

where x is the input image and w_i_ is the weight assigned to the individual response of the *ith* network, P_i_(x). We use a heuristic search of the optimal values of the weights applied to the individual networks. The weights are randomly initialized and iteratively evolve so that the overall performance of the resulting ensemble improves. We solve this optimization problem by minimizing the overall error of the ensemble using the Tree-structured Parzen Estimator (TPE)^39^, an improved version of Sequential Model-based Algorithm Configuration (SMAC). Briefly, TPE constructs the surrogate function *p*(Y|w), a probabilistic model of the objective function which maps the hyperparameters w to a probability of the score Y, by appyling the Bayes’ theorem. Instead of modeling the surrogate *p*(Y|w) directly, TPE models *p*(w|Y) and *p*(Y), where *p*(w|Y) is modeled by two non-parametric density functions based on observations w.

The final predicted label is based on the optimal decision rule, where the predicted class is that which has the highest probability.

**Figure 2:**
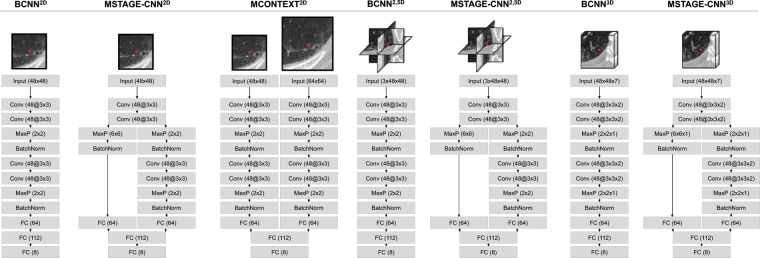
Architectures of the CNNs included in the proposed ensemble. Each network is fed with a 2D, 2.5D or 3D ROI extracted from a sample point.

The proposed ensemble is comprised of seven Convolutional Neural Networks: three 2D CNNs, two 2.5D CNNs and two 3D CNN, as detailed in Fig. 2.

Radiographic ILA patterns are mainly characterized by local texture patterns in CT images. In order to capture these local texture patterns, our baseline architecture uses relative small kernels in convolutional layers in order not to introduce non-local information in the receptive field. We also reduced the number of pooling layers through the network compared to standard CNNs to avoid spatial information loss. In view of these considerations, and considering that shallow CNNs do not have enough discriminative power while too deep architectures are computationally expensive to train and can easily overfit the training set, our baseline architecture (BCNN2D) for the classification of ILA radiographic patterns in HRCT images is a deeper variant of the well-known LeNet configuration, where each convolutional layer with 5 × 5 kernels are replaced with two layers with 3 × 3 kernels. We also introduced a batch normalization layer after each pooling operation to accelerate the network training and improve the overall performance.

In order to introduce architectures that exploit contextual information into the ensemble, we designed the multi-stage and multi-context versions of the baseline architecture (MSTAGE-CNN2D and MCONTEXT-CNN2D respectively). Multi-stage networks exploit contextual information by branching middle-level features from the CNN and concatenating them with the last features before being fed into the classification step, so that the network has richer representation of the input image with different scales of the receptive field. This configuration combines, at the classification step, high-level features from the latter stage with global information, and low-level features that represent local structures from the image so that the loss of spatial relationship between global features is avoided. Multi-context networks also add a secondary path to the architecture that parallel processes a larger scale input image so that the network simultaneously learns features from the input image at multiple scales.

To take advantage of spatial information, we designed the respective 2,5D and 3D versions of the baseline and multi-stage networks previously presented (yielding the BCNN2.5D, MSTAGE-CNN2.5D, BCNN3D and MSTAGE-CNN3D configurations), though the 2,5D and 3D multi-context versions were discarded due to their inadequate performance and convergence. 2,5D networks explore two-and-a-half dimensional (2,5D) representation of the image, learning features and capturing information from the three orthogonal planes, namely axial, sagittal and coronal slices. It has been shown that a two-and-a-half dimensional representation of pulmonary segments is optimally cost effective and has been claimed to be more suitable compared to more complex architectures^26^. A natural extension of 2,5D architectures are 3D CNNs which have the ability to model and capture volumetric contextual information around the voxel to classify.

All the architectures of the CNNs that comprise the proposed ensemble are described in details in Fig. 2.

The training of the CNNs is based on an optimization problem that minimizes a loss function. In this work we use Adam optimizer to minimize the L2-regularized categorical cross entropy.

### Overfitting prevention

Deep architectures, which involve a large number of parameters, easily overfit the training data. In this work we apply four different techniques to prevent and reduce the overfitting.

In order to increase the number of training samples and to consider variability through samples from the same class, we used two approaches to augment the training data by modeling both geometric and physiological transformations. Geometric transformations on training samples belonging to the classes with less training data (i.e. ground glass, nodular, linear scar and subpleural line) were used, combining rotation, horizontal and vertical shifts and flips and shearing. Additionally, in all samples of the training data, we also performed a domain-specific data augmentation to model the differences of tissue density depending on the level of inspiration, which is frequently needed when estimating pulmonary diseases in CT images^40^. In this work, we implemented a new approach to augment the training data modeling variations in lung density and volume due to differences on inspiration levels among patients in order to be robust and relatively independent of the respiratory state of the lungs at acquisition time. Our approach is based on the so-called sponge model of the lung^41^ which assumes mass preservation over the lung respiratory cycle causing that a proportional decrease in lung volume would yield an equally proportional increase in density. We have modeled random lung volume variations of ±100 milliliters (mL) —${\mathscr{U}}(-\mathrm{1000,}\,\mathrm{1000)}$—, corresponding to mild fluctuations in inspiratory lung volume.

On the other hand, we have also applied a regularization of the loss function for overfitting prevention. L2 regularization penalizes the square magnitude of the parameters (*w*) in the loss function by adding the term 1/2*λw*^2^, where *λ* is the regularization strength. This method penalizes sharp changes in the parameters, preferring soft ones. Dropout technique^42^ was also used to prevent the overfitting by randomly dropping units with a given probability *p* from the network during training. Finally, we also employed early stopping technique, where the training stage ends before overfitting process begins. The training finishes when the error on the validation set (validation loss) does not decrease for a preset iteration epochs (patience).

## Experiments and Results

### Experimental setup

#### Implementation details

All individual networks from the proposed ensemble method were trained on Regions of Interest (ROIs) extracted around the manually labeled points. The size of the ROIs was selected in accordance with each network architecture, always ensuring the coverage of a whole secondary pulmonary lobe (functional and anatomical unit of the lung), leading to ROIs of 30 mm^2^ mean size for the 48 × 48 axial patches. As derived from Fig. 2, we extracted two-dimensional ROIs from the axial plane for 2D-CNNs, while two-and-a-half-dimensional ROIs (from axial, sagittal and coronal planes) and three-dimensional ROIs were extracted for 2.5D-CNNs and 3D-CNNs respectively. Of the two available images per scan, the dataset was built with ROIs extracted from the CT images reconstructed with the B50 filter.

The only pre-processing performed on the dataset was normalizing the attenuation by subtracting the mean and dividing by the standard deviation tissue density computed on training and validation sets to normalize the image ROIs.

The framework used to implement the proposed method was based on Theano and Lasagne libraries using a PC with GPU GeForce GTX TITAN X Pascal 12GB, CPU Intel Core i7 3.6 GHz and 32GB of RAM.

#### Evaluation

To evaluate all proposed and state-of-the-art methods for the classification of ILA patterns, we constructed a train-validation-test scheme. For radiographic tissue classes with 800 or more training points, we randomly selected 600 samples (maximum of 75%) to be part of the training set, while for those classes with less than 800 points (i.e. ground glass, nodular, linear scar and subpleural line), 75% of the points were randomly separated for the training set and augmented with geometric transformations until all classes had 600 points in order to build an equally distributed training set. These training data were then split using a 10-fold cross validation scheme leading to create the final training and validation datasets, and all the CNNs were trained and evaluated 10 times according to the 10-fold cross validation split. For all tissue classes, the remaining samples not considered in this selection were defined as the test set (minimum of 25%). The training of the models was carried out on the training sets, and the validation sets were used for hyper-parameter tuning. The final performance evaluations of the proposed and state-of-the-art methods were carried out on the test sets.

Additionally, and for further validation, we performed an additional experiment with a train-test split at patient level, creating a completely independent test set without overlap between samples from the same CT scan in both the training and the new independent test sets. This experiment allows us to check the repeatability of our results at the subject level assuming that disease presence within a subject is somehow correlated between lung regions. From the 254 CT scans described in Database section, we randomly selected 5 patients for testing ensuring that all eight tissue classes under study were represented, and the rest of the scans were used for training, leading to 4800 training points and 2330 samples for testing.

We employed five point-metrics to evaluate and compare the proposed and baseline classification methods To deal with the skewed class distribution of the test dataset and to equalize the importance of each class, we computed the point-metric for each class, and estimated their unweighted average. We considered the sensitivity (SN), specificity (SP), geometric mean (GM) and balanced accuracy (BA), defined as:

2 $${\rm{SN}}=\frac{{\rm{TP}}}{{\rm{TP}}+{\rm{FN}}}\,{\rm{SP}}=\frac{{\rm{TN}}}{{\rm{TN}}+{\rm{FP}}}\,{\rm{GM}}=\sqrt{{\rm{TP}}\,\ast \,{\rm{TN}}}\,{\rm{BA}}=\frac{{\rm{SN}}+{\rm{SP}}}{2},$$

where TP, FP, TN and FN detone the number of true positives, false positives, true negatives and false negatives respectively.

### Validation results

In the following subsections we describe a set of experiments performed to validate the proposed method. First, we compared the proposed ensemble to the individual networks. Second, we evaluated the generalizability of the method when it is applied to data with characteristics different than those of the training set. Next, we performed an overall analysis of the system’s performance with an independent test dataset. Finally we performed a comparison of our techniques with previous studies, as well as an experiment to investigate the performance of our method when it is applied to the full lungs with sliding ROIs.

#### Ensemble of models

We first compared the performance of individual networks to the proposed multi-model ensemble. To identify the optimal architectures of individual networks we experimented with different configurations, resulting in the architectures shown in Fig. 2. To optimize the training procedure we also experimented with different hyper-parameters, but for purposes of comparative evaluation, we used the same parameter set for all individual CNNs of the ensemble. In Table 2 we summarize these hyper-parameters tuned via cross-validation. The weights w assigned to the individual response of each network of the ensemble (Eq. 1) were also optimized via cross validation.

**Table 2:** Fine-tuned hyperparameters.

Hyper-parameter	Value
Optimizer	Adam updates *η* = 0.001, *β*_1_ = 0.9, *β*_2_ = 0.999, *ε* = 10e-08
L2 regularization strength	0.0025
Patience (early stopping)	30
Dropout rate	0.5
Batch size	128

**Figure 3:**
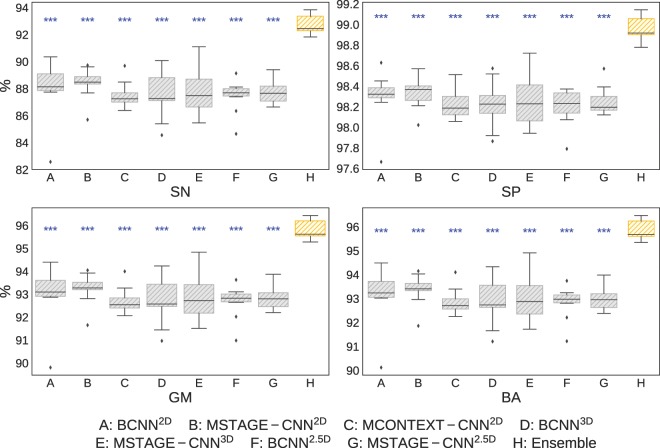
Classification performance of each individual models (gray) and combined in the ensemble (orange). The two-samples t-test to assess the statistical significance of the improvement in performance of the ensemble compared to the individual networks are represented with blue asterisks (***two-sample t-test: *p*-value < 0.001). SN: sensitivity, SP: specificity, GM: geometric mean, BA: balanced accuracy.

Figure 3 compares the proposed ensemble with the individual networks performance by giving the unweighted average along all classes for each evaluation metric. It can be seen that the ensemble achieved a significantly (two-sample t-test: *p*-value < 0.001) better performance compared to isolated models by combining its individual responses, outperforming in terms of all considered metrics the individual CNNs, and reducing the standard deviation of the mean of each metric.

Table 3 demonstrates the effect of using different strategies to aggregate the individual networks to build the ensemble, including weighting and non-weighting methods. For weighting methods, we used different optimization algorithms to optimize the weights. Minimizing the overall error of the ensemble using cross-validation, we concluded that all the methods yielded roughly the same performance, with TPE providing the best results, followed by Bayesian Optimization (BO) and Random Search (RS). When Majority Voting rule (MVR) was used to combine the individual responses it also obtained a higher performance compared to individual models, but all weighting methods scored better than the latter.

**Table 3:** Classification performance using different aggregation strategies to build the ensemble.

Ensemble strategy		SN(%)	SP(%)	GM(%)	BA(%)
Weighting method	RS	92.39 (±0.55)	98.91 (±0.10)	95.60 (±0.33)	95.65 (±0.32)
	TPE	92.51 (±0.62)	98.93 (±0.10)	95.67 (±0.37)	95.72 (±0.36)
	BO	92.47 (±0.58)	98.93 (±0.09)	95.64 (±0.34)	95.70 (±0.33)
Non-weighting method	MVR	91.81 (±0.87)	98.83 (±0.12)	95.25 (±0.51)	95.32 (±0.49)

SN: sensitivity; SP: specificity; GM: geometric mean;

BA: balanced accuracy.

As shown in Table 4, there was considerable variation between the learned weights of each of the individual networks using the different optimization algorithms, although all performed similar (Table 3). This may be due to the fact that all algorithms find different but equivalent local minima and close to the same global minima.

**Table 4:** Optimized weights.

Weigths	Optimization Algorithm		
	RS	TPE	BO
w_1_: BCNN2D	0.468	0.682	0.745
w_2_: MSTAGE-CNN2D	0.635	0.979	1.000
w_3_: MCONTEXT-CNN2D	0.285	0.367	0.447
w_4_: BCNN3D	0.630	0.781	0.849
w_5_: MSTAGE-CNN3D	0.690	0.98	1.000
w_6_: BCNN2.5D	0.145	0.373	0.389
w_7_: MSTAGE-CNN2.5D	0.645	0.902	1.000

#### Evaluation of generalization capability

Because CNNs learn features from their training data and it is important to determine if they are generalizable to data with characteristics that are different than those of the training dataset. For CT imaging data this may include diverse acquisition protocols including different CT scans vendors, reconstruction kernels, and other imaging characteristics.

The database used for training the individual networks from the ensemble is composed of different scans from diverse vendors and models, which leads to a non-homogeneous dataset, and likely improves generalizability. We sough to further explore the generalizability of our approach, specifically relating to reconstruction kernel, by taking the advantage of the fact that we were able to extract two different ROIs corresponding to two different reconstruction kernels for each point in the dataset (B35 and B50 filters).

**Figure 4:**
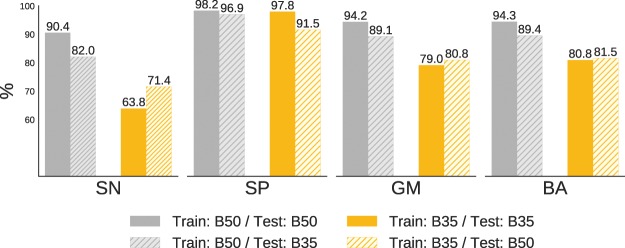
Evaluation of the proposed ensemble optimized with TPE when trained and tested on datasets with images reconstructed with different kernels (B35 and B50), with respect to the evaluation metrics SN (sensitivity), SP (specificity), GM (geometric mean) and BA (balanced accuracy).

As shown in Fig. 4, we achieved the higher performance when training and testing were carried out on images reconstructed with the B50 filter, corresponding to the sharp reconstruction kernel. These results are in accordance with the clinical routine, where the use of sharper images are preferred when performing visual identification and quantification of interstitial diseases. Additionally, it is also shown the generalization capability of the proposed method when the networks are trained and tested on images reconstructed with different kernels.

#### Analysis of the ensemble performance

**Figure 5:**
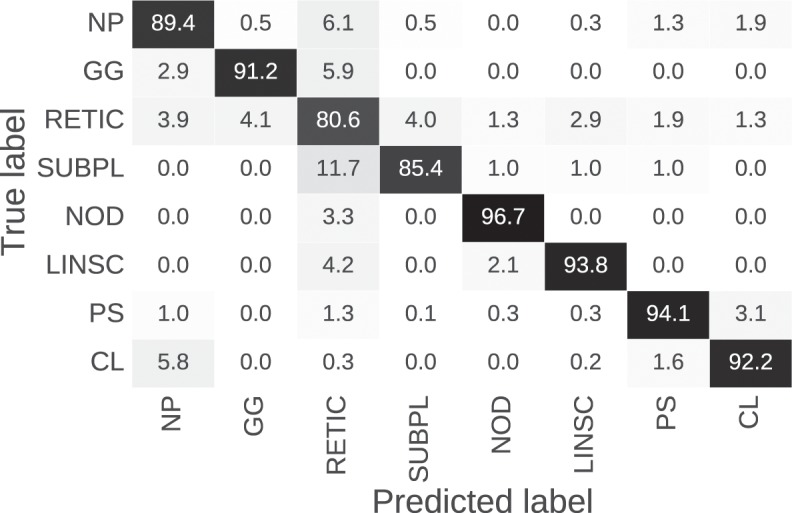
Normalized confusion matrix of the proposed ensemble optimized with TPE for the eight tissue classes under study.

Figure 5 shows the normalized confusion matrix of the proposed ensemble optimized with TPE. As shown, the misclassifications generally occurred within interstitial group, where misclassified reticular samples were mainly confused with subpleural line and nodular patterns. Misclassifications also occurred between normal parenchyma and centrilobular emphysema. Detailed numerical outcomes of each class are presented in Table 5.

**Table 5:** Detailed performance of the proposed method.

Group	Class	#Samples		Results			
		CV Train	Test	SN(%)	SP(%)	GM(%)	BA(%)
HEALTHY	Normal parenchyma (NP)	600	23096	0.8937	0.9649	0.9286	0.9293
INTERSTITIAL	Ground glass (GG)	600	34	0.9118	0.9907	0.9504	0.9512
	Reticular (RETIC)	600	4809	0.8064	0.9501	0.8753	0.8782
	Nodular (NOD)	600	30	0.9667	0.9977	0.9820	0.9822
	Linear scar (LINSC)	600	48	0.9375	0.9936	0.9652	0.9656
	Subpleural line (SUBPL)	600	103	0.8544	0.9907	0.9200	0.9225
EMPHYSEMA	Paraseptal emphysema (PS)	600	3245	0.9405	0.9861	0.9630	0.9633
	Centrilobular emphysema (CL)	600	3013	0.9220	0.9810	0.9510	0.9515
	Avg/Total	4800	34378	0.9041	0.9818	0.9420	0.9430

SN: sensitivity; SP: specificity; GM: geometric mean; BA: balanced accuracy.

In addition, an evaluation of the proposed method on a subject independent test set with respect to the training set showed a sensitivity (SN) of 88.9%, specificity (SP) of 98.4%, geometric mean (GM) of 93.5% and balanced accuracy (BA) of 93.61%. The results of our solution when training using each sample independently or using only samples from the same subjects are comparable. Our interpretation is that the lung damage is a process that affects the secondary lobule and is somehow independent across different secondary lobules. Therefore training and testing could be done using samples from the same subject as long as they belong to different secondary lobules as it is the case in our training set.

Understanding and interpreting the results obtained with deep learning techniques is of paramount importance. Grad-CAM technique is a valuable manner to better understand and interpret what trained deep models have learned^43^. We mapped the activation maps corresponding to one of our proposed two-dimensional networks (BCNN2D). Activation maps highlight the regions for each sample that most influence the predictions. We observed that the activation maps consistently select the location of the lesions (see Supplementary Figure S2).

#### Comparison with the state-of-the-art methods

Table 6 compares the performance of the state-of-the-art methods to the proposed ensemble of CNNs (ECNN). The first row corresponds to a method based on local histogram hand-crafted features (LH)^10^, while the rest correspond to methods that use CNNs for feature extraction and classification, including the well-known GoogleNet^44^ (57 convolutional layers) and VGG-16-Net^45^ (13 convolutional layers) networks. Both networks were trained from scratch on our dataset, and pretrained on ImageNet and fine-tuned on our data (GoogleNet-P and VGG-P). Also shown the results of the CNN proposed by Anthimopoulos *et al*.^31^, which focused on detecting patterns of interstitial lung diseases from 2D patches, as well as those obtained with the architecture proposed by Kim GB *et al*.^33^, which considers an image patch of 20 × 20 pixels. We independently implemented all of the methods and trained and tested them using the same data and framework. Table 5 also presents the performance of all individual networks that constitute the ensemble (last seven rows).

**Table 6:** Comparison of the proposed ensemble with state-of-the-art methods.

Method	SN(%)	SP(%)	GM(%)	BA(%)
LH^10^	0.6888	0.9376	0.7976	0.8132
VGG	0.6241	0.9319	0.7565	0.7780
VGG-P	0.6553	0.9703	0.7727	0.8128
GoogleNet-P	0.6930	0.9264	0.7943	0.8097
GoogleNet	0.6256	0.9341	0.7608	0.7798
VGG-EF	0.7762	0.9633	0.8647	0.8697
VGG-LT	0.8148	0.9678	0.8880	0.8913
Kim *et al*.^33^	0.3759	0.9027	0.5825	0.6393
Anthimopoulos et al.^31^	0.7687	0.9657	0.8567	0.8672
**ECNN**	**0.9041**	**0.9818**	**0.9420**	**0.9430**
BCNN2D	0.8615	0.9730	0.9152	0.9172
MSTAGE-CNN2D	0.8552	0.9740	0.9125	0.9146
MCONTEXT-CNN2D	0.8621	0.9766	0.9171	0.9194
BCNN2.5D	0.8426	0.9748	0.9051	0.9087
MSTAGE-CNN2.5D	0.8776	0.9754	0.9246	0.9265
BCNN3D	0.8271	0.9650	0.8917	0.8960
MSTAGE-CNN3D	0.8335	0.9727	0.8993	0.9031

The first group of methods (first seven rows) presents the results of the state-of-the-art and the proposed ensemble methods. The second group presents the results of the individual models.

SN: sensitivity; SP: specificity; GM: geometric mean; BA: balanced accuracy.

Additionally, and for a more extended comparison, we have also implemented two different ensembles of VGG-16 networks. The ensembles are composed by nine VGG networks, each of them having as an input a single image patch including the seven axial images which comprise the 3D volume as well as the sagittal and coronal views. We used the VGG networks pretrained on ImageNet as a feature extractor. We then combine the features using both early and late fusion. For the early fusion (VGG-EF), we fused the features with a single multilayer perceptron trained on our own dataset. Conversely, for the late fusion (VGG-LF), the information is fused at the decision level, where the final fully connected layers of the nine VGG networks were fine tuned on our data independently and the predictions were subsequently fused using a soft voting rule.

**Figure 6:**
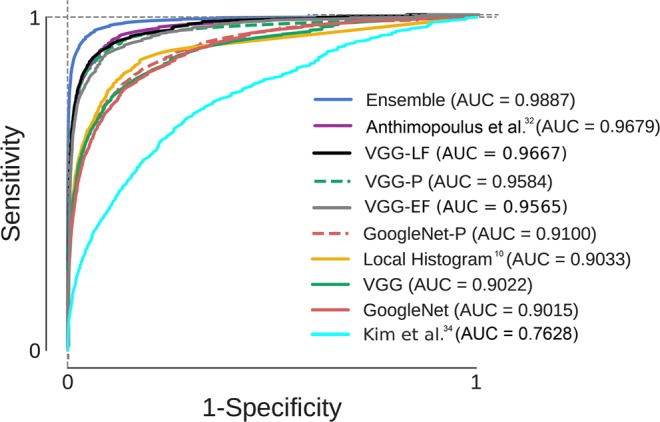
ROC analysis for the proposed ensemble and eight sate-of-the-art methods. The ROC curves represent the macro-average over all classes. The area under the curve (AUC) values are also given for each ROC. VGG-P and GoogleNet-P stands for the pretrained versions on ImageNet of VGG-Net and GoogleNet architectures. VGG-LF and VGG-EF stands for the ensemble of VGG networks using late and early fusion respectively.

**Figure 7:**
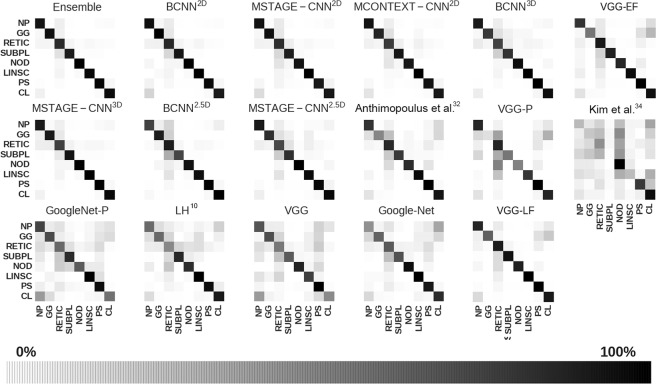
Normalized confusion matrices of the proposed ensemble, individual networks that make up the ensemble and state-of-the art methods.

In general, our proposed ensemble method, outperformed the other techniques. For example, our approach had a 28% higher BA than the method that uses hand-crafted features. In addition, compared with the rest of the methods based on CNNs, our ensemble method outperformed the best of them by 7.6% in terms of BA. We also found that very deep CNNs such as GoogleNet easily overfit the data when they are not trained in very large datasets, yielding poor performance on the test set. The ensambles of VGG networks which process multi-view and multi-dimensional data do not outperformed the porposed method. The macro-average ROC curves for each method are shown in Fig. 6 and demonstrated that the proposed method achieved the highest area under the curve (AUC). Finally, for comparison, in Fig. 7, the confusion matrices of the proposed method, the individual networks that compose the ensemble and state-of-the-art are all shown.

#### Visual evaluation

In order to complete the validation from a clinical point of view and to extend the analysis from isolated ROIs, we performed a visual evaluation of the proposed method. For these analyses, we computed the full-lung classification of twenty CT scans from COPDGene cohort at different stages of disease severity and ILA patterns.

Full-lung classification was carried out at a fixed sampling grid with spacing 5 × 5 × 5 voxels, and the rest of the voxels were classified using a nearest-neighbor interpolator. The time needed for the proposed method to process an average-sized entire scan (700 slices) using a sampling grid of 10 × 10 × 10 voxels was 3,5 minutes, while the corresponding time needed when a sampling grid of 5 × 5 × 5 is used was 25 minutes, using the previously described hardware.

For visual scoring, two experts inspected the classification results of twenty full lung classifications performed by two methods: the proposed ensemble of CNNs, and, for comparision, a local histogram-based method^10^, which has previously shown to identify interstitial patterns with both clinical and generic associations^46^. The scoring was done in a blinded manner, and based on the consensus of the two reviewers as to the goodness of the method to classify each of the eight interstitial. Additionally, the performance in terms of the spatial consistency of the resulting labeled lung and the classification precision on the hilum, which is an area prone to errors due to its anatomical complexity, were assessed. Visual scoring of the results of the subtypes classification was done using a range from 0 to 10 according to the degree of under- and over-estimation of the extent of the tissue class, where 0 means significant under estimation, 5 means neither under estimation nor over estimation of the subtype, and 10 represents a high over estimation. Visual scores for spatial consistency and precision on the hilum range from 5 to 10, where 5 means high or good spatial consistency and hilar precision and 10 low spatial consistency and hilar precision.

**Figure 8:**
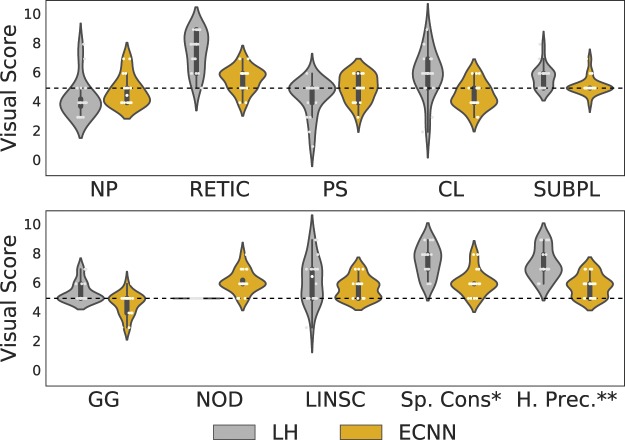
Visual score for twenty full-lung classification results obtained with the proposed ECNN and LH methods. *Spatial Consistency; **Hilar Precision.

Table 7 and Fig. 8 summarize the visual scores for both methods. Table 7 reports the Mean Absolute Error (MAE), defined as the distance to 5, for all the inspected aspects (subtypes and overall considerations), and the mean of overestimation (scores > 5) and underestimation (scores < 5) for all subtypes. Additionally, for reference, the Mean Percentage Volume (MPV) of each subtype under study was measured for the twenty cases. For each case the subtype percentage volume was estimated as the mean classified volume provided by both LH and ECNN methods. Although for some subtypes the local-histogram based method exhibits a better behaviour in terms of extent definition, the proposed ensemble of CNNs is significantly better than those obtained with the local histogram-based method in terms of Mean MAE for all the subtypes (two-sample t-test, *p*-value < 0.001). It can be also observed that for the subtypes with higher presence in our test population (defined as MPV ≥ 5%) the ECNN performs better than the local-histogram approach.

**Table 7:** Visual assessment of the proposed ensemble and local histogram-based method. ECNN stands for the proposed Ensemble of Convolutional Networks method.

	MPV	MAE		Over.		Under.	
	*(mean* ± *sd)*	LH	ECNN	LH	ECNN	LH	ECNN
NP	73.2 ± 10.9	1.25	0.80	6.00	5.60	3.89	4.37
RETIC	10.0 ± 5.6	2.45	0.80	7.45	5.77	5.00	4.75
PS	3.2 ± 1.6	0.70	0.70	5.07	5.50	4.32	4.46
CLE	6.5 ± 9.2	1.45	0.80	6.33	5.33	4.17	4.23
SUBPL	1.5 ± 0.6	0.75	0.25	5.75	5.21	5.00	4.94
GG	1.2 ± 1.0	0.40	0.50	5.40	5.07	5.00	4.52
NOD	2.5 ± 3.8	0.00	1.15	5.00	6.15	5.00	5.00
LINSC	1.9 ± 0.9	1.40	0.70	6.37	5.70	4.78	5.00
Sp. Cons.*	—	2.6	1.10	—	—	—	—
H. Prec.**	—	2.35	0.85	—	—	—	—
Mean	—	1.33	0.76	5.92	5.54	4.64	4.66

*Spatial Consistency; **Hilar Precision.

*MPV*: Mean Percentage Volume, *MAE*: Mean Absolute Error, *Over*.: Overestimation, *Under*.: Underestimation.

LH stands for Local Histogram.

**Figure 9:**
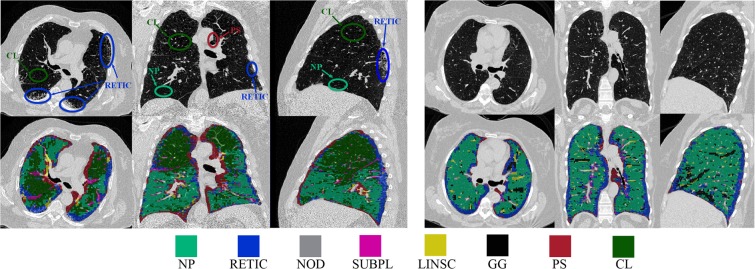
Automated full lung classification results of two CT scans with different ILA subtypes and stage of disease severity. The first row of the left figure shows several ground truth areas.

As can be seen in Fig. 9, qualitative review of the technique’s performance suggests that while the overall performance of the approach was excellent, there were a few systematic errors. These include the classification of linear bronchovascular bundles as linear scar (LINSC), the misclassification of proximal bronchovascular bundles viewed in cross-section as nodular (NOD), and the identification of motion artifact related to the fissures and diaphragm as ground glass (GG), all of which are errors that can be easily removed via masking. Reticular (RETIC) areas were also sometimes misclassified as Nodular (NOD) pattern. This effect was previously described, and is also observed in the confusion matrix (Fig. 5).

#### Clinical validation

For further clinical validation we wanted to test if the proposed method is directly associated with the visually determined score of the presence of ILA. We hypothesized that the proposed method can determine the binary classification of a patient presenting ILA using as benchmark the visual identification of ILA.

The visual assessment of CT scans for ILA has been carried out following the previously described procedure^1^. All individual’s CT scans were visually analyzed and determined to have ILA if there were nondependent ground-glass or reticular abnormalities, diffuse centrilobular nodularity, nonemphysematous cysts, honeycombing, or traction bronchiectasis affecting more than 5% of any lung zone.

**Figure 10:**
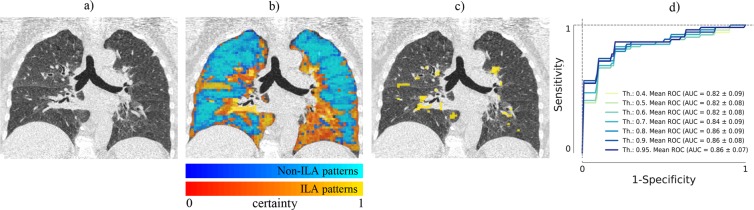
(**a**) Original image; (**b**) Certainty map given by the proposed method for non-ILA labeled voxels (blue) and ILA labeled voxels (yellow); (**c**) Lung area affected by interstitial patterns with certainty greater than 95%; (**d**) ROC curves using several thresholds for the detection of visually defined ILA.

We defined a quantitative ILA score as the percentage of the lung area affected by any interstitial pattern, namely ground glass, reticular, nodular, linear scar and sub pleural line where only the voxels with greater than a determined certainty threshold were considered. The certainty of a voxel was defined based on the probability given by the proposed ensemble of CNNs and associated to the predicted label for this voxel (see example in Fig. 10a-c).

Receiver operating characteristic curves and the area under the curve were generated using logistic regression via cross-validation for the prediction of those individuals with visually identified presence of ILA.

CT scans from 114 subjects, that were not included in the training set, were evaluated (52 with visually defined ILA and 62 without ILA). Overall the proposed method had an area under the receiver operating characteristic (ROC) curve of 0.863 ± 0.067 for the detection of visually defined ILA when using a certainty threshold of 95%. For lower certainty thresholds the classifier performance slightly decreased as shown in Fig. 10d.

## Discussion and Conclusions

In this work, we proposed a novel method to detect and classify radiographic patterns of ILD at an early stage in CT images, considering eight radiographic classes of lung tissue, including normal tissue, five interstitial features subtypes and two emphysematous classes. Specifically, we proposed a multi-model ensemble of deep CNNs. The ensemble is comprised of seven CNNs, incorporating 2D, 2.5D and 3D networks. To this end, each individual network was trained from scratch on our database, and then the outputs of the networks are summed up in a weighted manner and combined to form the overall output of the ensemble. Both the CNNs and the weights of the ensemble are trained via cross-validation. The resulting ensemble achieved a higher performance compared to each of the individual models, proving the potential of combining the responses of various classifiers.

The augmentation in learning enabled by utilizing different CNN architectures has been scarcely used in medical imaging but shows promising results^47^. Our proposal combines deeper and shallower representations with different input dimensionality. The optimized ensemble weights show that each of the single models contributes to the final classification. Although there is not a single local minima for the ensemble, the different optimization approaches produce comparable results in terms of accuracy. This might indicate some degree of redundancy in the prediction provided by each ensemble. Further studies could include the minimum set of information that is needed to achieve similar performance.

Previous studies have shown the advantage of using 2.5D and 3D architectures encoding more spatial contextual information, and therefore producing representations with higher discrimination capability^48,49^. Our study proves that the combination of different input data representations (2D, 2.5D and 3D) is important, given that the proposed ensemble improves the performance with respect to the isolated models.

Our study suggests that the proposed method can be employed to identify radiographic patterns of ILD at an early stage. In our test studies, which involve a large dataset of 34378 samples, the performance of our method showed greater area under the ROC curve (AUC) than state-of-the-art architectures commonly used by the computer vision community. Our method also outperforms two specific architectures of CNN to detect ILD patterns^31,33^, as well as a simpler approach to objectively identified ILA based on density histogram features^10^.

Additionally, we have shown the generalization capability of our method to be applied to large cohorts of patients with data reconstructed with different kernels. We performed intra-kernel and inter-kernel studies in which the training and test images were reconstructed with the same and different kernels respectively, and to the best of our knowledge, this is the first work that comprises images with different reconstruction algorithms and that has studied and verified the generalization capability of a method for automated classification of lung tissue.

We also performed a quantitative experiment where the method was tested on a completely independent test set separating the training and testing dataset at the patient level. This experiment allowed us to ensure the repeatability of results and generalization of the proposed method.

To confirm the clinical feasibility of the proposed method we performed two experiments including a visual evaluation of the proposed method and a clinical validation where we tested the association between the method and the visually determined score of the presence of ILA. For the visual evaluation, two experts visually evaluated and scored by consensus twenty full-lung classification results. The results of the clinical visual assessment confirmed the statistically significant (*p*-value < 0.001) superior performance of the proposed method against previous work based on local histogram features that has recently shown to provide insightful clinical and genetic associations^46^. Our visual evaluation highlights the difficulty of assessing interstitial subtypes with low prevalence. It is worth noting that the subtypes for which our method obtained lower accuracy in terms of visual scoring, are the ones that correspond to the cases with the lowest number of training and testing samples that may influence the ability of their CNN approach to learn their unique characteristics. We expect that the inclusion of more training data for these subtypes could alleviate this effect.

Regarding the ability of the method for detecting visually defined ILA, the experiment carried out on 114 subjects showed that estimation of ILA patterns in high certainty areas seems to better predict the visual assessment of ILA, suggesting the importance of the prediction probability.

In summary, we have demonstrated that the proposed ensemble of CNNs, in spite of the intrinsic difficulty of the problem due to the subtle nature of the disease, is able to identify radiographic patterns of early parenchymal lung disease that have been previously shown to be associated with prognosis and clinical outcomes. It has also been shown that our method can identify individuals with interstitial abnormalities, but further study is needed to determine the association between the response of the proposed method and other clinical outcomes including mortality.

## Supplementary information

Supplementary  Information

## Data Availability

CT cases used in this work are part of COPDGene study and can be obtained through https://www.ncbi.nlm.nih.gov/projects/gap/cgi-bin/study.cgi?study_id = phs000179.v5.p2dbGap.
