# Supplementary material for: Classification of Interstitial Lung Abnormality Patterns with an Ensemble of Deep Convolutional Neural Networks
Source: Sci Rep. 2020 Jan 15;10:338. doi: 10.1038/s41598-019-56989-5 (PMC6962320; doi:10.1038/s41598-019-56989-5)

Classification of Interstitial Lung Abnormality Patterns with an Ensemble of Deep Convolutional Neural Networks

David Bermejo-Peláez[1,*], Samuel Y. Ash[2], George R. Washko[2], Raúl San José Estépar[2,+], María J. Ledesma Carbayo[1,+]

[1]Biomedical Image Technologies, ETSI Telecomunicación, Universidad Politécnica de Madrid & CIBER-BBN, Madrid, Spain
[2]Applied Chest Imaging Laboratory, Brigham and Women's Hospital, Boston, Massachusetts, United States of America

*Corresponding author. Email: david.bermejo@upm.es
+RSJ and MJLC contributed equally to this work

Table S1. Institutional Review Board Approval Documentation COPDGene.

**Institutional Review Board Approval Documentation COPDGene**		
**Participating Center**	**Institution Title for Review Board**	**Protocol Number**
National Jewish Health	National Jewish IRB	HS-1883a
Brigham and Women's Hospital	Partners Human Research Committee	2007-P-000554/2; BWH
Baylor College of Medicine	Institutional Review Board for Baylor College of Medicine and Affiliated Hospitals	H-22209
Michael E. DeBakey VAMC	Institutional Review Board for Baylor College of Medicine and Affiliated Hospitals	H-22202
Columbia University Medical Center	Columbia University Medical Center IRB	IRB-AAAC9324
Duke University Medical Center	The Duke University Health System Institutional Review Board for Clinical Investigations (DUHS IRB)	Pro00004464
Johns Hopkins University	Johns Hopkins Medicine Institutional Review Boards (JHM IRB)	NA_00011524
Los Angeles Biomedical Research Institute	The John F. Wolf, MD Human Subjects Committee of Harbor-UCLA Medical Center	12756-01
Morehouse School of Medicine	Morehouse School of Medicine Institutional Review Board	07-1029
Temple University	Temple University Office for Human Subjects Protections Institutional Review Board	11369
University of Alabama at Birmingham	The University of Alabama at Birmingham Institutional Review Board for Human Use	FO70712014
University of California, San Diego	University of California, San Diego Human Research Protections Program	070876
University of Iowa	The University of Iowa Human Subjects Office	200710717
Ann Arbor VA	VA Ann Arbor Healthcare System IRB	PCC 2008-110732
University of Minnesota	University of Minnesota Research Subjects' Protection Programs (RSPP)	0801M24949
University of Pittsburgh	University of Pittsburgh Institutional Review Board	PRO07120059
University of Texas Health Sciences Center at San Antonio	UT Health Science Center San Antonio Institutional Review Board	HSC20070644H
Health Partners Research Foundation	Health Partners Research Foundation Institutional Review Board	07-127
University of Michigan	Medical School Institutional Review Board (IRBMED)	HUM00014973
Minneapolis VA Medical Center	Minneapolis VAMC IRB	4128-A
Reliant Clinic	Institutional Review Board/Research Review Committee Saint Vincent Hospital – Fallon Clinic – Fallon Community Health Plan	1143

Figure S2. Activation maps generated by BCNN^{2D} network. Correct label appears above the original image patch. Predicted label appears above the activation maps. Activation maps corresponding to normal parenchyma (NP) focused on the entire image patch, almost uniformly, as there is not any radiological sign of disease. Activation maps corresponding to linear scar (LINSC), centrilobular emphysema (CL), subpleural line (SUBPL) and nodular pattern (NOD) focused on those areas that define each disease subtype and lesion location. For parastatal emphysema (PSE) regions the network focused on both low attenuation areas representing emphysema and pleural regions.

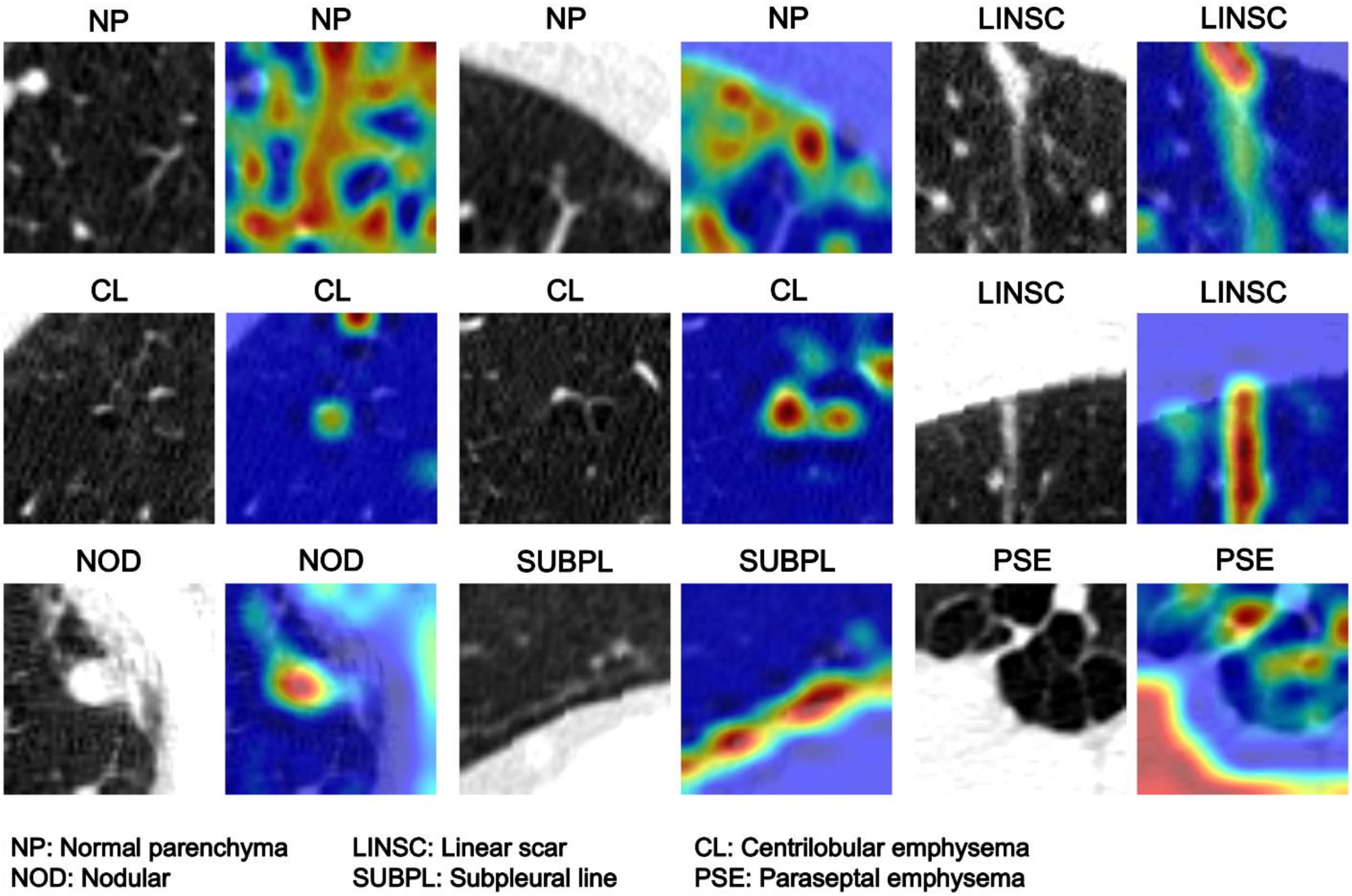

Supplement: Supplementary file 1 — Supplementary Information [file 41598_2019_56989_MOESM1_ESM.pdf]
